# Central role of carotid body chemoreceptors in disordered breathing and cardiorenal dysfunction in chronic heart failure

**DOI:** 10.3389/fphys.2014.00438

**Published:** 2014-11-24

**Authors:** Noah J. Marcus, Rodrigo Del Rio, Harold D. Schultz

**Affiliations:** ^1^Department of Cellular and Integrative Physiology, University of Nebraska Medical CenterOmaha, NE, USA; ^2^Laboratory of Cardiorespiratory Control, Center of Biomedical Research, Universidad Autónoma de ChileSantiago, Chile

**Keywords:** carotid body chemoreceptors, Cheyne–Stokes respiration, sympathetic nervous system, heart failure, cardiorenal syndrome

## Abstract

Oscillatory breathing (OB) patterns are observed in pre-term infants, patients with cardio-renal impairment, and in otherwise healthy humans exposed to high altitude. Enhanced carotid body (CB) chemoreflex sensitivity is common to all of these populations and is thought to contribute to these abnormal patterns by destabilizing the respiratory control system. OB patterns in chronic heart failure (CHF) patients are associated with greater levels of tonic and chemoreflex-evoked sympathetic nerve activity (SNA), which is associated with greater morbidity and poor prognosis. Enhanced chemoreflex drive may contribute to tonic elevations in SNA by strengthening the relationship between respiratory and sympathetic neural outflow. Elimination of CB afferents in experimental models of CHF has been shown to reduce OB, respiratory-sympathetic coupling, and renal SNA, and to improve autonomic balance in the heart. The CB chemoreceptors may play an important role in progression of CHF by contributing to respiratory instability and OB, which in turn further exacerbates tonic and chemoreflex-evoked increases in SNA to the heart and kidney.

## Introduction

Abnormal oscillatory breathing (OB) patterns are frequently observed in diverse populations, including infants born prematurely (Copeman et al., 1964), patients with heart failure (Ponikowski et al., 1999), or end stage renal disease (Hanly and Pierrato, 2001), and in otherwise healthy humans who travel to high altitude (Lahiri et al., 1983). These abnormal breathing patterns most commonly occur during non-REM sleep when chemical control of breathing predominates; however, some heart failure patients exhibit OB during waking hours as well (Brack et al., 2007). OB is characterized by oscillations in tidal volume and/or respiratory frequency and is thought to occur as a result of physiological or environmental challenges that de-stabilize the respiratory control system. These challenges may include alterations in arterial blood gases and pH (decreased P_a_O_2_, decreased P_a_CO_2_, and increased pH), circulatory delay and reductions in systemic oxygen transport, and enhancement of respiratory chemoreflex function (Fanfulla et al., 1998). The etiology of OB is diverse; however a significant body of research indicates that enhanced chemoreflex sensitivity is a common element of most types of OB (Lahiri et al., 1983; Ponikowski et al., 1999; Al-Matary et al., 2004; Nock et al., 2004; Hering et al., 2007).

## Chemoreflex sensitivity and disordered breathing in heart failure

Cheyne–Stokes respiration (CSR), a form of OB in which oscillations in tidal volume are separated by apneic episodes, is highly prevalent in patients with chronic heart failure (CHF) (Mortara et al., 1997; Ponikowski et al., 1999; Giannoni et al., 2008). CSR is associated with increased morbidity and mortality, and decreased quality of life in this population (Hanly and Zuberi-Khokhar, 1996; Lanfranchi et al., 1999; Brack et al., 2007; Carmona-Bernal et al., 2008). Accumulating evidence suggests that enhanced central and/or peripheral chemoreflex sensitivity (Javaheri, 1999; Narkiewicz et al., 1999; Giannoni et al., 2008) as well as persistent hyperventilation/hypocapnia (Naughton et al., 1993; Fanfulla et al., 1998) contribute to the pathogenesis of CSR by causing instability of the respiratory control system (Naughton et al., 1993; Lorenzi-Filho et al., 1999, 2005; Pinna et al., 2000). The significance of the relationship between chemosensitivity and CSR is further underscored by the finding that high peripheral chemosensitivity is independently associated with poor prognosis and higher mortality risk in CHF patients but not in comparable CHF patients with low chemosensitivity (Ponikowski et al., 1999, 2001).

Numerous studies indicate that carotid body (CB) chemoreceptor-mediated responses to hypoxia and hypercapnia are augmented in CHF (Wilcox et al., 1993; Chua et al., 1996, 1997; Javaheri, 1999; Ponikowski and Banasiak, 2001; Ciarka et al., 2006; Giannoni et al., 2008). In a group of 60 CHF patients, approximately 60% had increased CB chemoreflex sensitivity (Giannoni et al., 2008). Most importantly, patients without augmented chemosensitivity did not exhibit CSR, and the incidence of CSR progressively increased with enhancement of the CB chemoreflex. In other studies, deactivation of CB chemoreceptors with transient hyperoxia, or pharmacological attenuation of chemosensitivity with dihydrocodeine or acetazolamide significantly reduced central apnea incidence in CHF patients (Ponikowski et al., 1999; Fontana et al., 2011). These findings indicate an important relationship between CSR or cyclical breathing patterns and enhanced CB chemoreflex sensitivity.

Recent studies in animal models of CHF have further delineated the role of the CB chemoreceptors in OB. Studies from our laboratory have demonstrated enhanced ventilatory, sympathetic nerve, and carotid sinus nerve responses to isocapnic hypoxia as well as a tonic increase in resting afferent chemoreceptor discharge during normoxia in both rabbit and rat models of heart failure (Sun et al., 1999a,b; Li et al., 2005; Del Rio et al., 2013b; Haack et al., 2014; Marcus et al., 2014a). These increases in CB chemoreceptor activity coincide with an increase in measures of OB and the development of CHF (Marcus and Schultz, 2011). Denervation of the CB chemoreceptors (CBD) by CB ablation after the development of CHF results in abolition of chemoreflex responses, reduction of resting ventilation and sympathetic nerve activity (SNA), and reduction of apnea/hypopnea frequency and respiratory variability (Del Rio et al., 2013b; Marcus et al., 2014a). In other studies, pharmacologic attenuation of CB chemoreceptor activity with Simvastatin or an inhibitor of hydrogen sulfide production had similar efficacy in reducing apnea/hypopnea frequency and respiratory variability (Del Rio et al., 2013a; Haack et al., 2014).

Ablation of CB afferent activity in the aforementioned studies (Del Rio et al., 2013b; Marcus et al., 2014a) resulted in significant reductions in resting ventilation, which in turn would be expected to increase resting PaCO_2_. CHF-CBD rabbits exhibited significant hypoventilation relative to normal animals for up to 9 days post CBD, the endpoint of the study (Marcus et al., 2014a). CHF-CBD rats exhibited hypoventilation compared to the ventilatory parameters obtained in normal animals when measured 2 days post denervation, but no hypoventilation was found at 14 weeks post CBD (Del Rio et al., 2013b). Thus, the salutary effect of CBD to stabilize the respiratory pattern in CHF could stem from an increase in P_a_CO_2_ above the apneic threshold, at least in the short-term, but abrogation of the elevated ventilatory loop gain mediated by the CB chemoreflex is likely to play an important role in reestablishing respiratory stability in CHF in the long-term.

Resting ventilation and sympathetic outflow are increased in CHF (Naughton et al., 1993; van de Borne et al., 1998). In our studies, CBD-reduced resting sympathetic outflow as well as ventilation, indicating that CB chemoreceptors play an important role in the tonic increases in both of these parameters in CHF. Central neural coupling between respiratory and sympathetic neural drive has been described in the literature (Haselton and Guyenet, 1989). It is possible that the elevated tonic input from CB chemoreceptors exacerbates respiratory-sympathetic coupling to account in part for their marked increase in CHF patients.

## Respiratory-sympathetic coupling in heart failure

It is well-known that sympathetic discharge is actively modulated by respiration (Adrian et al., 1932; Haselton and Guyenet, 1989), and a growing body of evidence indicates that this modulatory influence may be altered in several different pathological states. Evidence of enhanced respiratory-sympathetic coupling has been found in three different animal models of hypertension (Zoccal et al., 2008; Simms et al., 2009; Toney et al., 2010) with differing etiologies (spontaneously hypertensive rat-SHR, Ang II/salt, and chronic intermittent hypoxia-CIH). Interestingly, in two of these models (SHR and CIH), enhanced CB chemoreflex sensitivity and tonic CB chemoreceptor afferent input to the brain stem have been shown to play a seminal role in mediating increased SNA and the development of hypertension (Fletcher et al., 1992; Peng et al., 2003; Del Rio et al., 2010; Marcus et al., 2010; Tan et al., 2010; Abdala et al., 2012). Furthermore, sympathetic drive increases in tandem with respiratory neural output after exposure to CIH (Zoccal et al., 2008). No studies have examined CB chemoreflex tone in the Ang II/salt model, however Ang II has been shown to play a role in enhancing CB chemosensitivity (Li et al., 2006), and thus it is plausible that tonic CB chemoreceptor input is elevated in this model as well. Evidence from these studies suggests that enhanced afferent activity arising from the CBs promotes respiratory-sympathetic coupling that in turn perpetuates sympathetic over activity.

**Figure 1:**
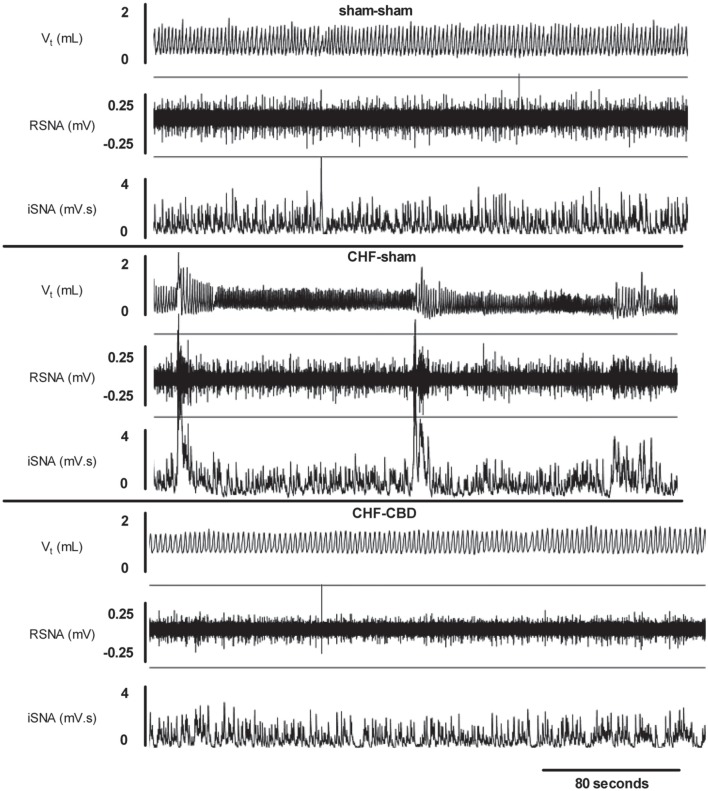
**Respiratory-sympathetic coupling in CHF**. Oscillatory breathing patterns were apparent in CHF animals (middle panel) that were accompanied by concomitant oscillations in renal sympathetic nerve activity (RSNA). Respiratory and RSNA oscillations were not observed in CHF animals after carotid body denervation (bottom panel). CHF-chronic heart failure, CBD-carotid body denervation, V_t_-tidal volume, RSNA-renal sympathetic nerve activity, iSNA-integrated renal sympathetic nerve activity. Reproduced with permission from Marcus et al. (2014a).

Recent work from our lab (Figure 1) has shown that respiratory-sympathetic coupling is enhanced in CHF, and that the enhanced coupling coincides with sensitization of the CB chemoreflex (Marcus et al., 2014a). Furthermore, we demonstrated that respiratory-sympathetic coupling in CHF is critically dependent on the CB since it was markedly reduced or abolished after CBD (Marcus et al., 2014a). Taken together, these findings strongly suggest a central role for enhanced tonic CB chemoreceptor drive in the development of respiratory-sympathetic coupling in disease conditions characterized by autonomic imbalance and abnormal respiratory rhythms.

The mechanisms underpinning the relationship of CB chemoreflex drive to respiratory-sympathetic coupling in CHF are still unclear. A plausible hypothesis is that the entrainment between the respiratory and sympathetic neural drive may result from alterations in the neurons integrating CB afferents and initiating respiratory rhythm and sympathetic outflow in the brainstem. Indeed, there is evidence that CIH-induced sympatho-excitation results in an increase in the strength of the excitatory synapses at the level of the nucleus of the solitary tract, the paraventricular nucleus, and the rostral medulla (Kc et al., 2010; Kline, 2010; Silva and Schreihofer, 2011; Costa-Silva et al., 2012). Enhanced respiratory-sympathetic coupling is of major relevance in CHF patients in which hyperventilation is common, and in which frequent respiratory oscillations occur during CSR (Figure 1). Previous investigators have observed surges in SNA during the hyperpneic phase of CSR (Leung et al., 2006) which may be indicative of enhanced respiratory-sympathetic coupling, and which likely has important impact on downstream targets such as the heart and kidneys.

## Role of enhanced chemoreflex sensitivity and disordered breathing in cardiac and renal dysfunction in heart failure

In CHF patients, renal dysfunction is common and is associated with poor prognosis (Bock and Gottlieb, 2010). Development of renal dysfunction in CHF is particularly ominous because it can precipitate further decline in cardiac function, initiating a downward spiral of deteriorating cardiac and renal function, known as cardiorenal syndrome. While the etiology of cardiorenal syndrome is diverse, excessive sympathetic activation, volume retention and venous congestion, renal ischemia secondary to reductions in renal perfusion, and neuro-hormonal activation are thought to play central roles (Bock and Gottlieb, 2010). Tonic chemoreflex activation in CHF may contribute to cardiorenal syndrome by increasing sympathetic stimulation of the heart (Xing et al., 2014) and kidneys (Sun et al., 1999a) leading to increases in peripheral vascular resistance and myocardial oxygen demand, increases in sodium and water retention, and activation of the renin-angiotensin system. In addition, the development of OB mediated by enhanced CB chemoreflex sensitivity may further exacerbate renal ischemia by eliciting additional chemoreflex-evoked renal vasoconstriction in addition to episodic hypoxemia (Figure 1).

Under normal circumstances, CB chemoreflex activation elicits a reduction in renal blood flow and glomerular filtration rate that is mediated by renal sympathetic nerves (Karim et al., 1987). In CHF, tonic elevations in renal SNA mediate sustained reductions in renal blood flow and alterations in angiotensin signaling (Clayton et al., 2011). Our preliminary findings indicate that the reduction in renal blood flow to CB chemoreflex activation is markedly accentuated in CHF animals. Further, CBD in CHF animals reduces renal SNA, increases renal blood flow, and decreases markers of renal injury and fibrosis (Marcus et al., 2014b), in addition to the reduction in disordered breathing and improvement in cardiac function mentioned previously (Marcus et al., 2014a). These findings suggest that tonic CB chemoreflex activation in CHF may contribute to renal pathology in part by its influence on sympathetic outflow (Hering et al., 2007) to the heart and kidneys (Sun et al., 1999b; Xing et al., 2014). In addition to the influence of tonic CB chemoreflex activation on resting renal SNA, additional surges in SNA may be superimposed by episodic hypoxemia associated with apneic episodes during sleep (van de Borne et al., 1998), augmented by an enhanced CB chemoreceptor sensitivity to hypoxia in CHF (Marcus et al., 2014a). This notion is supported by evidence from studies in clinical populations (Ryan et al., 2005).

Normalization of abnormal breathing patterns in CHF patients with continuous positive airway pressure (CPAP) or adaptive servo-ventilation (ASV) is associated with reduced tonic levels of sympathetic activation (Ryan et al., 2005), improved cardiac function, improved renal function, and improved prognosis (Koyama et al., 2011; Yoshihisa et al., 2011; Kasai et al., 2013; Owada et al., 2013). These improvements may be due to secondary effects of CPAP or ASV treatments to improve cardiac function via direct mechanical effects of pressure support ventilation on the heart (Takama and Kurabayashi, 2011), however they also likely reflect the reduction in CB chemoreflex sensitivity (Spicuzza et al., 2006), and consequent reduction in CB chemoreflex-mediated sleep disordered breathing and sympathoexcitation (Naughton et al., 1995; Despas et al., 2009). Our findings in an animal model of CHF support this notion of the functional consequences of enhanced respiratory-sympathetic coupling in CHF mediated by the CB. The reduction of disordered breathing patterns with CBD was sufficient to reduce renal SNA, increase renal blood flow, and improve cardiac function (Marcus et al., 2014a,b) and survival (Del Rio et al., 2013b), independent of any confounding effects of pressure support ventilation used in the aforementioned clinical studies.

## Conclusion

**Figure 2:**
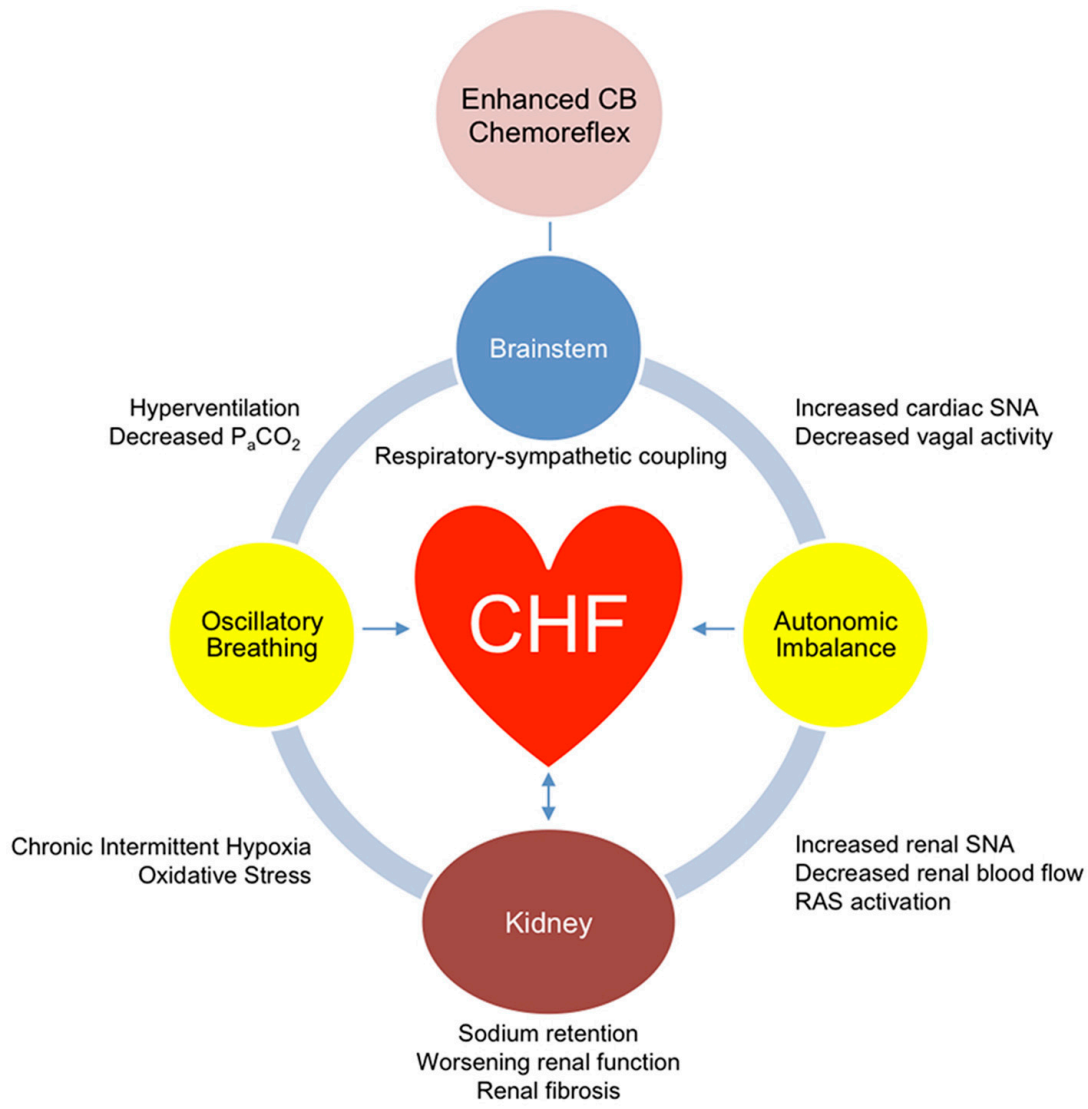
**Role of carotid body chemoreceptors in cardiac and renal dysfunction**. Enhanced tonic afferent activity from carotid body (CB) chemoreceptors drives neuronal activity in brainstem centers that integrate peripheral afferents and control respiratory and sympathetic neural outflow. Hyperventilation due to the enhanced CB chemoreflex activation precipitates oscillatory breathing, which exacerbates sympathetic activation through respiratory-sympathetic coupling, in addition to exposing the heart and kidneys to intermittent hypoxia and oxidative stress. The CB-mediated enhanced respiratory-sympathetic coupling results in increased sympathetic and decreased vagal efferent outflow to the heart, which over time worsens cardiac function and development of fibrosis. Similarly, CB-mediated increases in renal SNA cause reductions in renal perfusion and activation of the renin-angiotensin system (RAS), which over time lead to worsening renal function and development of fibrosis. The combined deleterious effects of CB-mediated respiratory-sympathetic coupling on the heart and kidney advances the cardiorenal syndrome.

Accumulating evidence suggests a critical role for the CB chemoreceptors in the etiology of several important pathophysiological aspects of CHF. CB chemoreceptors are a major driving force in the development of autonomic dysfunction and breathing abnormalities in CHF. Ablation of the CB chemoreceptors is sufficient to improve these parameters and leads to improved cardiac function (Marcus et al., 2014a) and survival (Del Rio et al., 2013b). The mechanisms by which the CB chemoreflex exacerbates cardiac deterioration and morbidity in CHF remain to be better elucidated, but disordered breathing, enhanced respiratory-sympathetic coupling, tonic and episodic increases in cardiac and renal SNA, and reductions in renal function likely play an important role (Figure 2). A case report published recently showed that unilateral CBD in a CHF patient resulted in modest improvements in autonomic function, cardiac function, and exercise tolerance, and reduced resting ventilation (Niewinski et al., 2013). This study supports findings from pre-clinical animal models and confirms the potential of CBD or other forms of CB modulation as a therapeutic option in CHF patients. Taken together, these findings suggest that CB-mediated disordered breathing and respiratory-sympathetic coupling in CHF plays an important role in the abnormalities of sympathetic outflow observed in CHF with negative clinical implications for cardiac and renal function (Marcus et al., 2014a,b).

### Conflict of interest statement

The authors declare that the research was conducted in the absence of any commercial or financial relationships that could be construed as a potential conflict of interest.
